# Bionic Modeling Study on the Landing Mechanism of Flapping Wing Robot Based on the Thoracic Legs of Purple Stem Beetle, *Sagra femorata*

**DOI:** 10.3390/biomimetics10010063

**Published:** 2025-01-17

**Authors:** Haozhe Feng, Junyi Shi, Huan Shen, Chuanyu Zhu, Haoming Wu, Lining Sun, Qian Wang, Chao Liu

**Affiliations:** 1 Robotics and Microsystems Center, College of Mechanical and Electrical Engineering, Soochow University, Suzhou 215021, China; fhz20040529@outlook.com (H.F.); 2262406040@stu.suda.edu.cn (J.S.); 2229403025@stu.suda.edu.cn (C.Z.); 2229403012@stu.suda.edu.cn (H.W.); lnsun@suda.edu.cn (L.S.); 2 College of Mechanical and Electrical Engineering, Nanjing University of Aeronautics and Astronautics, Nanjing 210016, China; shenhuan99@nuaa.edu.cn

**Keywords:** landing mechanism, flapping wing robot, purple stem beetle, bionic leg

## Abstract

Flapping wing micro aerial vehicles (FWMAVs) are recognized for their significant potential in military and civilian applications, such as military reconnaissance, environmental monitoring, and disaster rescue. However, the lack of takeoff and landing capabilities, particularly in landing behavior, greatly limits their adaptability to the environment during tasks. In this paper, the purple stem beetle (*Sagra femorata*), a natural flying insect, was chosen as the bionic research object. The three-dimensional reconstruction models of the beetle’s three thoracic legs were established, and the adhesive mechanism of the thoracic leg was analyzed. Then, a series of bionic design elements were extracted. On this basis, a hook-pad cooperation bionic deployable landing mechanism was designed, and mechanism motion, mechanical performance, and vibration performance were studied. Finally, the bionic landing mechanism model can land stably on various contact surfaces. The results of this research guide the stable landing capability of FWMAVs in challenging environments.

## 1. Introduction

Flapping wing micro aerial vehicles (FWMAVs) mimic the flight patterns of birds or insects, demonstrating high maneuverability, low-noise operation, and the ability to adapt effectively to varying natural conditions. These characteristics have shown immense potential for applications in various fields, such as military operations and environmental monitoring [1,2,3,4]. Compared to the traditional hard-contact landing methods of FWMAVs, designing flight landing actions with targeted strategies can alleviate the problems of insufficient landing capability and low environmental adaptability associated with traditional FWMAVs [5,6,7]. However, in contrast to natural flyers, which can land freely in complex natural environments, the environmental adaptability of current FWMAV landing mechanisms remains relatively low [8,9]. So, there are still challenges to be addressed before such flapping robots can be used in practice, and particularly their airflow disturbance [9], vision positioning [9], and surface condition (land on smooth surfaces, rough surfaces, cylindrical surfaces, and other irregular surfaces [9,10]) need to be improved.

Biomimetic technology, a method that studies the structures, functions, and mechanisms of organisms in nature to provide innovative solutions for the design of engineering technologies [11]. Natural flyers have developed excellent takeoff and landing capabilities through prolonged evolution to adapt to complex natural environments. This superior landing ability provides solutions for the design of landing mechanisms in FWMAVs. Researchers have discovered that insects’ unique perching behavior and leg structures grant them exceptional takeoff and landing capabilities in complex environments [12,13]. The thin secretion film on insect legs provides a large adhesive force, allowing insects to adhere to various contact surfaces [14,15]. Additionally, certain insects possess hooks or clamps that enable efficient landing and climbing on rough surfaces [16,17]. Furthermore, insects utilize suction organs [18] and sticky secretions [19], contributing to stable landing and adhesion. These distinctive structures offer inspiration for the design of landing mechanisms in micro aerial vehicles.

In recent years, significant progress has been made in developing FWMAVs inspired by bionic insects [20,21,22]. However, much of the research has focused on wing design and aerodynamic flight [23], while the bionic design of legs landing mechanisms, particularly those with highly adaptive landing capabilities, remains underexplored. The current strategies for FWMAV landing mechanisms primarily include the following approaches: landing on cylindrical surfaces [24,25] and rough surfaces [26] using mechanical claws and hooks, landing on smooth surfaces using adhesive pads and suction cups [27,28], as well as employing special landing forms such as electrostatic adhesion [29,30] and magnetic adhesion [31]. Researchers have carried out a series of development efforts based on these landing strategies. For example, a landing mechanism with a passive cushioning structure has been designed to improve landing stability [32]; however, it is limited to horizontal surface landings. Another design, inspired by birds, integrates landing and jumping actions using a hook-based grabbing mechanism [33], but it employs a single-grab type hook. Some researchers have developed an inverted landing mechanism for drones with dual self-locking capabilities inspired by bats [34], and a passive gripping mechanism for flying robots has also been designed [35]. However, these are limited to cylindrical surface landings. Therefore, most landing mechanisms of current FWMAVs can only land on a single type of contact surface, and this single type limits their overall practice in complex environments [36,37]. The cooperative landing mechanism with multiple types of contact surfaces needs to be further studied.

In this paper, the purple stem beetle (*Sagra femorata*) was selected as the biological prototype, and the beetle thoracic legs’ 3D models were established by micro-CT. Next, a hook-pad cooperation landing strategy was discovered. On this basis, a bionic FWMAV’s deployable landing model was designed, inspired by the thoracic legs of *S. femorata*. Then, the mechanism motion, mechanical performance, and vibration performance were studied. This work is expected to provide a technical reference for the landing mechanisms in FWMAVs.

## 2. Materials and Methods

### 2.1. Specimens

**Figure 1:**
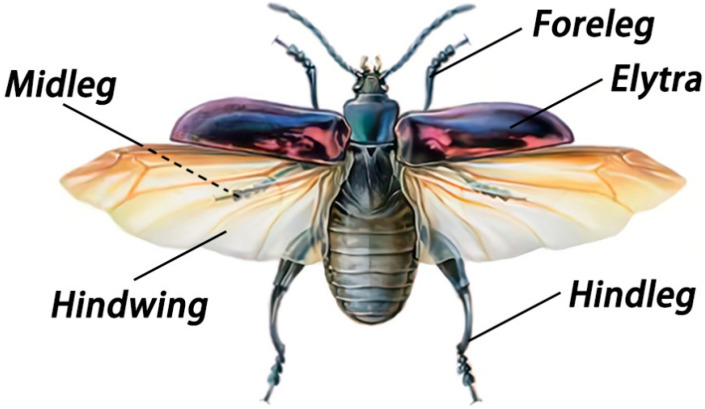
The moment of unfolding of *S. femorata* legs and wings.

The purple stem beetles, *S. femorata*, belonging to the order Coleoptera, collected from Liuzhou, Guangxi Province, China (Figure 1), were captured for use in this study. The body length of adult beetles is 20.0–25.0 mm, and the body width is 8.0–9.5 mm. Its body color typically ranges from purple to purplish-red. The foreleg and midleg of the adult beetle are relatively short, while the hindlegs are stronger. The ends of the thoracic legs are equipped with claws and suction pads. All thoracic legs used for experimental measurements were removed from freshly anaesthetized beetles and adhered strictly to established animal ethics standards.

### 2.2. The 3D Reconstruction of S. femorata Legs

Several *S. femorata* specimens were selected as subjects for morphological observation. The average length of the samples is 22.0 mm, and the average width is 8.3 mm. Thirty minutes before the start of the experiment, the foreleg, midleg, and hindleg on the same side of each specimen are removed using a scalpel and tweezers, respectively. The legs are carefully labeled and placed in an ultrasonic cleaner for disinfection and cleaning. The three processed legs are fixed in sequence at the center of the sample stage by using a glue gun, facilitating adjustments to the scanning light source and detector. The three-dimensional morphological details of *S. femorata* are observed using a high-resolution 3D analysis system (Xradia 620 Versa, ZEISS, Maple Grove, MN, USA).

## 3. Results

### 3.1. The 3D Reconstruction Model of S. femorata Thoracic Legs

**Figure 2:**
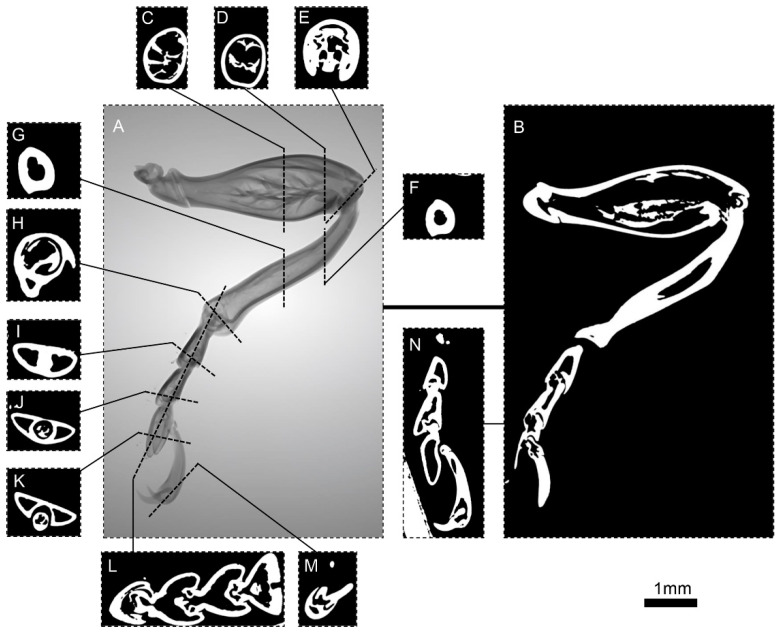
Micro-CT images of the foreleg of *S. femorata*. (**A**) Three-dimensional reconstruction of the foreleg surface. (**B**) Cross-sectional view of the foreleg. (**C**,**D**) Cross-sectional view of the foreleg femur. (**E**) Cross-sectional view at the joint between the femur and tibia. (**F**,**G**) Cross-sectional view of the foreleg tibia. (**H**) Cross-sectional view at the joint between the femur and tibia. (**I**–**K**) Cross-sectional view of the first, second, and third fore tarsomeres. (**L**) Sectional view of the fore tarsus. (**M**) Cross-sectional view of the fore tarsus. (**N**) Sectional view of the terminal actuator of the foreleg.

**Figure 3:**
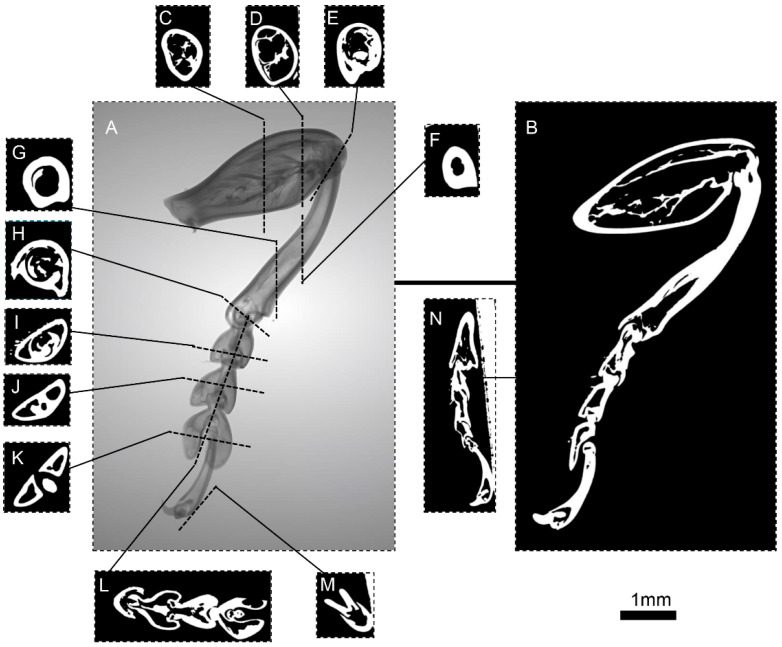
Micro-CT images of the midleg of *S. femorata*. (**A**) Three-dimensional reconstruction of the midleg surface. (**B**) Cross-sectional view of the midleg. (**C**,**D**) Cross-sectional view of the midleg femur. (**E**) Cross-sectional view at the joint between the femur and tibia. (**F**,**G**) Cross-sectional view of the midleg tibia. (**H**) Cross-sectional view at the joint between the femur and tibia. (**I**–**K**) Cross-sectional view of the first, second, and third mid tarsomeres. (**L**) Sectional view of the mid tarsus. (**M**) Cross-sectional view of the fore tarsus. (**N**) Sectional view of the terminal actuator of the midleg.

**Figure 4:**
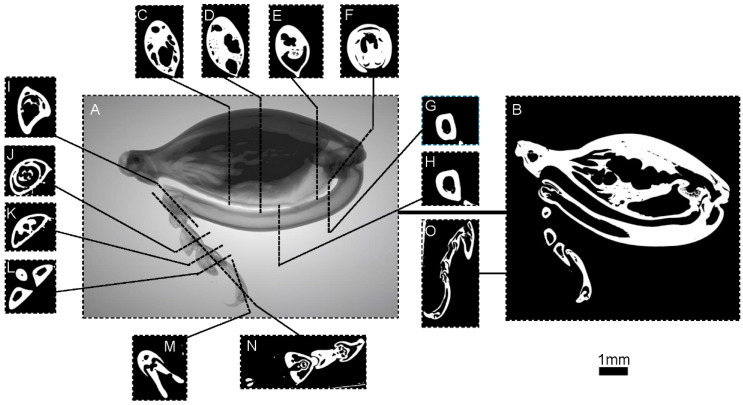
Micro-CT images of the hindleg of *S. femorata*. (**A**) Three-dimensional reconstruction of the hindleg surface. (**B**) Cross-sectional view of the hindleg. (**C**–**E**) Cross-sectional view of the hindleg femur. (**F**) Cross-sectional view at the joint between the femur and tibia. (**G**,**H**) Cross-sectional view of the hindleg tibia. (**I**) A cross-sectional view of the joint between the femur and tibia. (**J**–**L**) Cross-sectional view of the first, second, and third hind tarsomeres. (**M**) Cross-sectional view of the fore tarsus. (**N**) Sectional view of the hind tarsus. (**O**) Sectional view of the terminal actuator of the hindleg.

The 3D reconstruction images of the foreleg (Figure 2), midleg (Figure 3), and hindleg (Figure 4) of *S. femorata* were obtained by using a high-resolution 3D analysis system.

Figure 2 shows the overall micro-CT scan and cross-sectional images of *S. femorata* foreleg. The leg is connected to the body through the coxa and trochanter, allowing for multi-degree-of-freedom rotation. The femur is connected to the trochanter of *S. femorata* and is well developed, with a larger volume and a smooth contact surface. The tibia has a soft, elongated contact surface. The tarsus is pad-shaped with many bristles around it. The fore tarsus is slender and exhibits a bifurcated claw structure. As shown in Figure 2C,D, the radial cross-section of the femur is mostly elliptical, with the diameter peaking at the joint between the femur and the tibia (Figure 2E). This joint is encased at the end of the femur, forming a spherical structure with a concave shape, with the concave direction pointing towards the lower end of the femur. As shown in Figure 2F,G, the tibia has a hollow tubular structure with a smaller cross-sectional diameter. This lightweight and strong structure enables efficient and stable movement [38,39]. Figure 2H shows the joint between the tarsus and the tibia, which is similar in structure to the joint between the femur and tibia (Figure 2E), being spherical and concave. As shown in Figure 2I,J,L, the foreleg tarsus is divided into three segments. The segments are embedded within each other, with each tarsus segment connected at the central pad of the preceding one. All tarsal segments are hollow, functioning as one of the terminal effectors that allow *S. femorata* to land and take off freely in complex environments. Figure 2M,N shows the bifurcated claw (fore tarsus) embedded at the end of the third tarsus segment. This serves as another terminal effector for *S. femorata* during takeoff and landing. The claw structure provides additional friction for *S. femorata* during its movement process.

As shown in Figure 3, the structure of the midleg of *S. femorata* is similar to the foreleg, with an average length of approximately 13.49 mm, slightly longer than the foreleg (average length of approximately 12.62 mm). Figure 4 shows the hindleg of *S. femorata*, with an average length of approximately 19.47 mm. The radial dimensions of the femur in the hindleg are wider than the foreleg and midleg, with a higher proportion of muscle, providing the hindleg with greater muscular strength. The tarsus and foretarsus structures are similar to those of the foreleg and midleg, allowing the hindleg to have adhesive capability.

Table 1 lists the average length and diameter of each segment of the foreleg, midleg, and hindleg of *S. femorata*. From the average size, it can be observed that the femur is the longest segment in each leg, while the first tarsomere is the shortest. Regarding the tarsomeres, the length gradually increases with each successive segment. The average diameter shows that the femur’s diameter is approximately 2.3 times, 2.7 times, and 4.3 times the tibia for the foreleg, midleg, and hindleg, respectively. The average diameter of the tarsomeres is similar, about 0.26 mm, and smaller than the femur and tibia.

### 3.2. The Adhesive Mechanism of S. femorata Thoracic Legs

Analyzing the dimensions of each leg reveals that the hindleg is the largest, followed by the midleg, and the foreleg is the smallest. The average length of the hind femur is 1.77 times the midleg and 2.00 times the foreleg. The average diameter of the hind femur is 2.41 times the midleg and 3.10 times the foreleg. The differences between the midleg and foreleg are smaller; the midleg is 1.12 times longer and 1.28 times wider than the foreleg. In the tibia, the hind tibia’s length is 1.56 times the midleg and 1.68 times the foreleg, with the diameter being 1.52 times the midleg and 1.65 times the foreleg. The midleg’s tibia is 1.07 times longer and 1.08 times wider than the foreleg. The length differences among the three legs are small in the tarsus, with the midleg having the relatively longest tarsus. The first and second tarsomeres of the hindleg are smaller than those of the foreleg and midleg, while the third tarsomeres are similar. Regarding the average diameter of the tarsus, the diameters of the foreleg, midleg, and hindleg increase in a ratio of approximately 5:6:10. The length and average diameter distribution of the fore tarsus follows a similar pattern to the femur, decreasing in size from the hindleg to the midleg to the foreleg, with length ratios of approximately 1.37:1.14:1 and average diameter ratios of 2:1:1.

**Figure 5:**
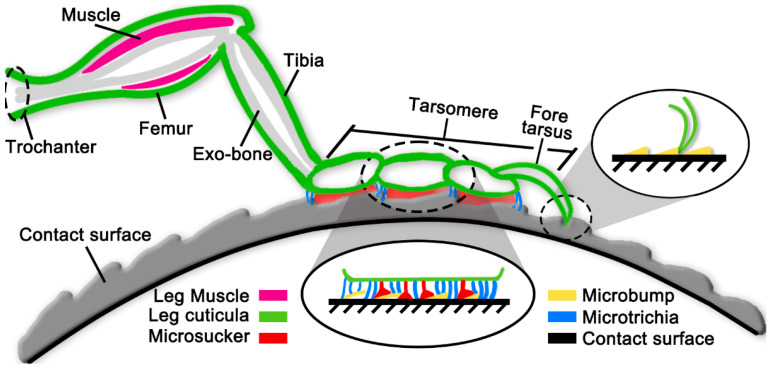
The hook-pad cooperation model (adhesive mechanism) of thoracic leg of *S. femorata*. After landing, *S. femorata* can flexibly apply the hook-pad cooperation strategy for different contact surfaces.

*S. femorata* uses the tarsus and fore tarsus (claws) for coordinated landing through hook-pad cooperation strategy, as shown in Figure 5. Regarding the adhesive mechanism (tarsus-pad), the tarsus of *S. femorata* consists of adhesive pads and bristles. The adhesive pad is composed of sticky bristles with a disc-shaped structure, and it is surrounded by bifurcated spatulate bristles, forming a bristle pad [40]. During the landing process of flying insects, some studies suggest that this adhesive pad structure acts like a suction cup on small, smooth surfaces [41]. The bristles, a common structure in most flying insects, enhance adhesion [12] on various landing surfaces by utilizing van der Waals forces, capillary action, and surface tension [13]. This helps them adapt to slightly uneven surfaces. Furthermore, the multi-bristle structure enhances friction and adhesion on smooth surfaces, stabilizing the insect’s posture on vertical or inclined surfaces, thus allowing it to adapt to diverse landing environments [42,43,44,45]. Regarding the gripping function (fore tarsus-claw), the bifurcated claws of *S. femorata* can grip rough surfaces, enabling it to land on such surfaces. In addition, the claws compensate for the adhesive force generated by the tarsus, achieving a coordinated landing through the hook-pad cooperation strategy (adhesive mechanism). This landing mechanism further improves *S. femorata*’s ability to land on complex surfaces [46].

Comparing the foreleg, midleg, and hindleg of *S. femorata*, the hindleg is the largest, while the tarsus is relatively short. This is likely related to its function in thrust during takeoff and for defense [47]. The longer tarsus in the foreleg and midleg is likely due to their use in adhesion and gripping during landing [40]. In conclusion, the coordinated mechanism of the three pairs of legs in *S. femorata* enables flexible landings in complex environments.

## 4. Discussion

### 4.1. Bionic Elements Extraction of S. femorata Thoracic Legs

Based on the establishment of the 3D reconstruction model of *S. femorata* thoracic legs and the analysis of the hook-pad cooperation strategy in the previous section, refer to the three-segment type thoracic legs, the multi-adsorption pad structure of the tarsomere, the hook mechanism of the froe tarsus, and the hollow tube structure of the whole leg; a bionic deployable landing leg model based on linkage mechanisms was designed. The extracted biomimetic elements of *S. femorata* thoracic legs are as follows:

- Multi-Tarsomere Structure

The legs of *S. femorata* consist of three tarsomeres and a fore tarsus. Flexible self-adaptive landing can be achieved through the relative rotation between the tarsomeres, allowing the legs to adapt to different contact surfaces (including curved and inclined surfaces).

- Hollow Tube Structure

The legs of *S. femorata* feature a hollow structure that is lightweight and high strength, improving its flight efficiency and mechanical properties. This structure allows energy storage and release during landing or rapid movement, enhancing the explosive power.

- Hook-pad cooperation Structure

The terminal part of *S. femorata* legs consists of tarsomeres and the fore tarsus (hooked claws). The fore tarsus (hooked claws) compensates for the adhesive force of the tarsal suction and setal pads, enabling synergistic adhesion on rough surfaces and producing a much stronger adhesion force than either component alone.

### 4.2. Design of Bionic Landing Mechanism

#### 4.2.1. Conceptual Design

**Figure 6:**
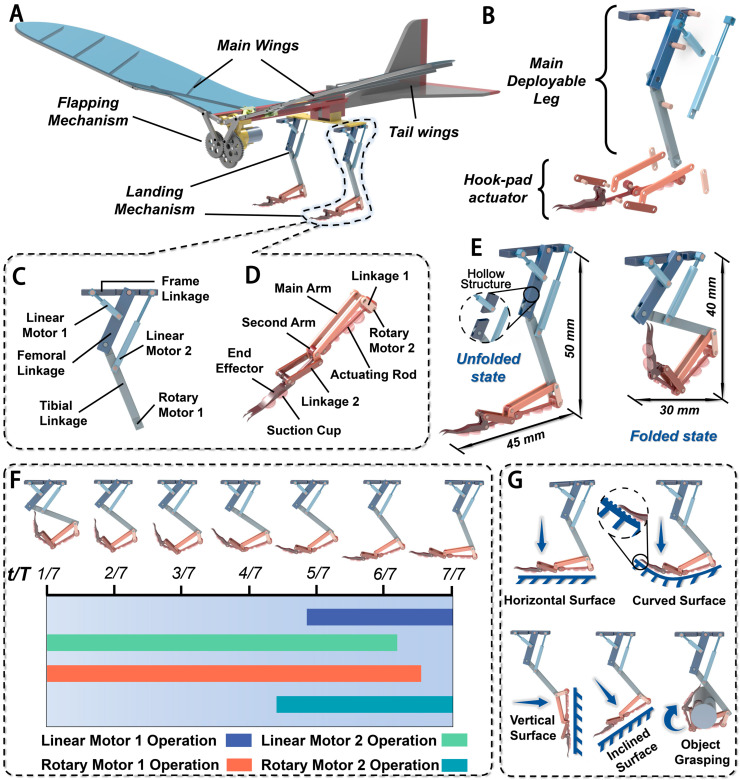
The designed linkage landing mechanism. (**A**) Landing mechanism installed on the FWMAV; (**B**) exploded view; (**C**) design of the main deployable leg; (**D**) design of the hook-pad actuator; (**E**) unfolded and folded states; (**F**) time variation and motor status during the unfolding process of the landing mechanism; (**G**) several various environments landing scheme.

The multi-tarsomere structure of *S. femorata*’s legs is mimicked, and a multi-linkage mechanism scheme is adopted to achieve the design of the bionic landing mechanism, as shown in Figure 6. The bionic landing mechanism primarily consists of two parts: the main deployable leg and the hook-pad actuator. It consists of the frame linkage, femoral linkage, tibial linkage, micro linear motor, micro rotary motor, main arm, second arm, end effector (bifurcated claw), linkage, actuating rod, and micro suction cups. The main deployable leg is connected to the hook-pad actuator through a hinged joint. The overall dimensions of the design are as follows: 50 mm × 45 mm in the deployed state and 40 mm × 30 mm in the folded state (details are shown in Figure 6E). The schematic diagram after installing the bionic landing mechanism to FWMAV developed by our team is shown in Figure 6A.

#### 4.2.2. The Design of the Main Deployable Leg

The femoral and tibial sections are crucial components of the bionic landing mechanism. A planar four-bar linkage mechanism is adopted to ensure efficient force transmission and structural stability. Due to the irregular shape of the actual *S. femorata*’s legs, which is not conducive to direct engineering design, certain effective simplifications were made. Specifically, the femoral and tibial sections are each composed of two linkages, with the femoral linkage being relatively thick and short and the tibial linkage being thin and long. The upper end of the femoral linkage is hinged to the frame linkage, while the lower end is hinged to the upper end of the tibial linkage.

To ensure the landing mechanism is lightweight while maintaining sufficient strength, two micro linear motors (1 and 2) are used. One motor is hinged to the middle of the femoral linkage and the other to the upper part of the tibial linkage. The upper ends of both motors are attached to the frame linkage. During the unfolding and folding of the landing mechanism, the two linear motors coordinate to drive the femoral and tibial linkages, ensuring the structure remains strong. Additionally, micro rotary motor 1 is mounted at the lower end of the tibial linkage to control the hook-pad actuator. The final design is shown in Figure 6B.

#### 4.2.3. The Design of the Hook-Pad Actuator

The tarsomeres of *S. femorata* contain numerous suction cups, and the entire tarsomere features a multi-tarsomere structure that provides good flexibility. To achieve contact surface adaptability and folding capability, the designed hook-pad actuator adopts a double-planar four-bar linkage mechanism. The final layout is shown in Figure 6C.

In this design, the main arm is hinged to the tibial linkage and is driven by micro rotary motor 1 located on the tibial linkage. One end of the second arm is hinged to the main arm, and the other end is hinged to the hook-pad actuator. Linkage 2 connects the second arm to the hook-pad actuator, while one end of linkage 1 is connected to micro motor 1. The actuating rod connects the second arm to linkage 1, with micro rotary motor 2 mounted at the hinge point. Suction cups are mounted on the actuating rod, second arm, and the lower part of the hook-pad actuator, with the size of the suction cups decreasing from the push rod to the second arm. The bending motion is achieved through the coordination of the two sets of planar four-bar linkages, ensuring the flexibility of the hook-pad actuator.

**Figure 7:**
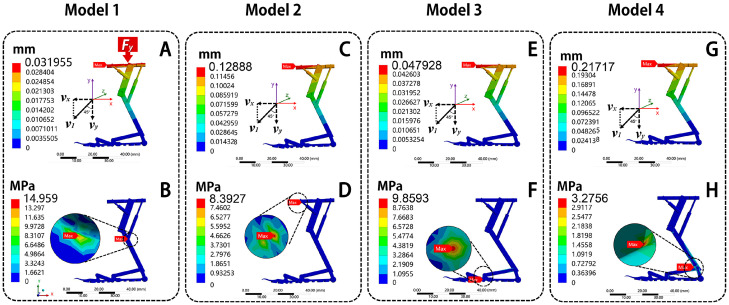
The diagrams of total deformation and equivalent stress of bionic deployable landing mechanism under uniform load. Total deformation: (**A**) Model 1, (**C**) Model 2, (**E**) Model 3, (**G**) Model 4; equivalent stress: (**B**) Model 1, (**D**) Model 2, (**F**) Model 3, (**H**) Model 4.

After that, all parts are assembled using various connectors (such as pins and sleeves) in Solidworks 2022 software. The final 3D assembly model is shown in Figure 7D,E.

### 4.3. The Mechanism Motion Simulation of Bionic Landing Mechanism

The motion simulation analysis of the bionic landing mechanism is completed using Solidworks 2022 software. The environmental temperature is 25°, and humidity is 40%. Linear motors are added to micro linear motors 1 and 2, and rotary motors are added to micro rotary motors 1 and 2 as driving components, with constant speed selected for motion. The time-related parameters for the motors are set as shown in Figure 6F. The simulation runs at 30 fps, with a minimum interval of 0.3, and the end time is set to 6 s. The mechanism’s operation process is shown in Figure 6F.

From Figure 6F, it can be observed that no interference between parts was detected, nor were there any redundant degrees of freedom. This demonstrates that the designed landing mechanism performs well, with a smooth and steady unfolding process. Additionally, due to the multi-linkage mechanism design, this landing mechanism exhibits higher adaptability than the current FWMAV landing technology (such as single grasping [48]; microtrichia adsorption [49]; magnetic adsorption [50]; and adhesive adsorption [51]) in complex landing environments. The motion of the mechanism is illustrated in Figure 6G, which can land on a horizontal surface, curved surface, vertical surface, inclined surface, and object grasping. And the hook-pad (Figure 6D) consists of three sections, which are composed of seven suction cups and the hook of the fore tarsus. The first section is 40 mm in length and includes a suction cup with a diameter of 2 mm and two suction cups with a diameter of 5 mm; the second section is 20 mm in length and includes two suction cups with a diameter of 5 mm; and the third section is 30 mm in length and includes a bifurcated hook with a length of 13 mm and two suction cups with a diameter of 5 mm. So, the landing structure combines grasping and adsorption, and the hook-pad cooperation landing strategy can avoid the limitation of the single landing contact surface of traditional FWMAV landings.

### 4.4. Mechanical Performance Analysis of Bionic Landing Mechanism

The structural static analysis was simulated on the bionic landing mechanism by Ansys Workbench 2022R2. Four comparative models were established to analyze the influence of material and structure changes on the mechanical properties. On the structure of the landing mechanism, two groups of models, solid and hollow, are set up. The hollow group is the bionic landing mechanism designed in this study (Figure 6E); the solid group is a solid model filled with a hollow structure. About the material of the landing mechanism, the material properties were assigned to the suction cups (polyurethane PU-60D, Tuode, Shanghai, China; elasticity modulus is 40.0 MPa, density is 1.2 g/cm^3^, Poisson’s ratio is 0.4, and the finite element type is thin plate element); the pins (aluminum alloy 7075, Wenext, Shenzhen, China; elasticity modulus is 7.17 × 10^4^ MPa, density is 2.81 g/cm^3^, Poisson’s ratio is 0.33, and the finite element type is rod element); the bifurcated claw and other linkages were made of two different materials, one is the 3D printing material-polylactic acid fiber (PLA), commonly used in the design of landing mechanisms [25,35,48] (PLA, Bambu Lab, Shenzhen, China; elasticity modulus is 2.5 GPa, density is 1.24 g/cm^3^, Poisson’s ratio is 0.33, and the finite element type is a rod element); and the other is carbon fiber [4,52] (carbon fiber T1000, Wenext, Shenzhen, China; elasticity modulus is 3.95 × 10^5^ MPa, density is 1.81 g/cm^3^, Poisson’s ratio is 0.2, and the finite element type is rod element).

**Figure 8:**
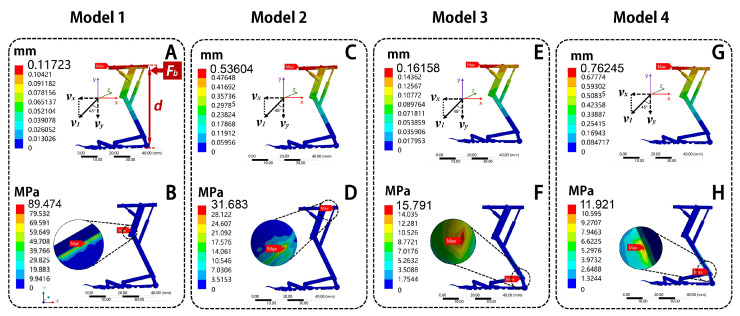
The diagrams of total deformation and equivalent stress of bionic deployable landing mechanism under bending moment. Total deformation: (**A**) Model 1, (**C**) Model 2, (**E**) Model 3, (**G**) Model 4; equivalent stress: (**B**) Model 1, (**D**) Model 2, (**F**) Model 3, (**H**) Model 4.

**Figure 9:**
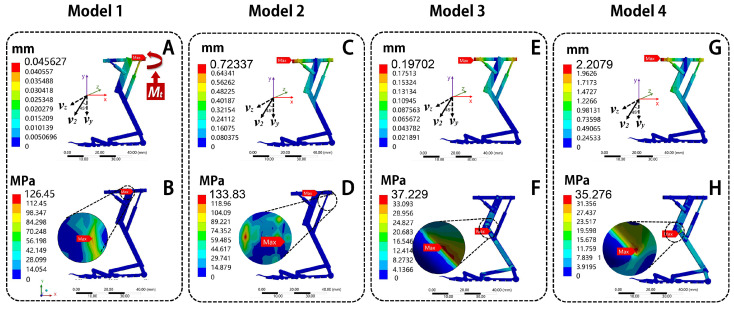
The diagrams of total deformation and equivalent stress of bionic deployable landing mechanism under twist moment. Total deformation: (**A**) Model 1, (**C**) Model 2, (**E**) Model 3, (**G**) Model 4; equivalent stress: (**B**) Model 1, (**D**) Model 2, (**F**) Model 3, (**H**) Model 4.

After that, four models were established: solid carbon fiber (Model 1), solid PLA (Model 2), hollow carbon fiber (Model 3), and hollow PLA (Model 4), as shown in Figure 7, Figure 8 and Figure 9. The grid was divided using a tetrahedral method, with a mesh size of 0.05 mm. After meshing, a total of 545,407 nodes and 284,041 elements in solid structure and a total of 559,271 nodes and 287,681 elements in hollow structure were generated, meeting the accuracy requirements. The load forms include uniform load, bending moment, and twist moment.

#### 4.4.1. Uniform Load

Uniform load is the primary load form that flying insects endure during landing, as the landing mechanism needs to resist the impact moment of touchdown. A normal uniform distributed load, denoted as *q*, is applied to the finite element model of the bionic landing mechanism, representing the pressure load it bears during landing. The calculation formula is as follows:

The vertical component of the landing velocity is:

(1) $$v_{y}=v_{1}\cdot sin\left(45{}^{\circ}\right)=1\;m/s\times 0.707\approx 0.707\;m/s$$

(2) $$F_{y}=\frac{m\cdot v_{y}}{\Delta t}=\frac{0.025\;kg\times 0.707\;m/s}{0.1\;s}\approx 0.177\;N$$

(3) $$q=\frac{F_{y}}{S}=\frac{0.177\;N}{7.5\times 10^{-7\;}m^{2}}=2360\;Pa$$

where $v_{1}$ is the landing speed of the flying robot [53], *v_y_* is the vertical velocity during landing, *F_y_* is the force acting on the landing mechanism model in the vertical direction, *m* is the mass of the flying robot, Δ*t* is the landing time, and *S* is the contact area. A fixed constraint is applied to the end position of the model, setting its degrees of freedom to zero.

The results are shown in Figure 7. From Figure 7A,C,E,G, the total deformation of the four models increases gradually from the suction cup to the frame linkage. The maximum deformation position is located on the frame linkage of all models, with deformation values of 0.032 mm, 0.12 mm, 0.048 mm, and 0.22 mm, respectively. From Figure 7B,D,F,H, the maximum stress of Model 1 is located at the connection between linear motor 2 and the tibial linkage, with the maximum stress of 14.96 MPa; the maximum stress of Model 2 is located on the frame linkage, with a maximum stress of 8.40 MPa; the maximum stress of Model 3 is located at the connection between the second arm and the actuating rod, with a maximum stress of 9.86 MPa; the maximum stress of Model 4 is located at the connection between the main arm and the femoral linkage, with a maximum stress of 3.28 MPa. Under the same uniform load, the total deformation of Model 1 is smallest, while Model 4 has the largest total deformation. The deformation of Model 1 is 75.20% smaller than Model 2, 33.32% smaller than Model 3, and 85.28% smaller than Model 4. About equivalent stress, the Model 4 is the smallest, while Model 1 has the largest equivalent stress. The maximum stress of Model 4 is 78.10% smaller than Model 1, 60.97% smaller than Model 2, and 66.78% smaller than Model 3.

It can be observed that the total deformation values of the four models are not much different. However, the equivalent stress of Model 4 is the smallest. It shows that the hollow PLA group can provide the best adaptability to compressive resistance performance.

#### 4.4.2. Bending Moment

A horizontal force *F_b_* is applied to top of the frame linkage in the bionic landing mechanism model. The horizontal component of the landing velocity (*v_x_*) and the bending moment *M_b_* exerted on the model is:

(4) $$v_{x}=v_{1}\cdot sin\left(45{}^{\circ}\right)=1\;m/s\times 0.707\approx 0.707\;m/s$$

(5) $$F_{b}=\frac{m\cdot v_{x}}{\Delta t}=\frac{0.025\;kg\times 0.707\;m/s}{0.1\;s}\approx 0.177\;N$$

(6) $$M_{b}=F_{b}\cdot d=0.177\;N\times 0.05\;m=0.00885\;N\cdot m$$

where *v*_1_ is the landing speed of the flying robot, *v_x_* is the horizontal velocity during landing, *F_b_* is the force acting on the landing mechanism model in the horizontal direction, *m* is the mass of the flying robot, Δ*t* is the landing time, and *d* is the top length of the landing mechanism. A fixed constraint is applied to the bottom position of the model, setting its degrees of freedom to zero.

The results are shown in Figure 8. From Figure 8A,C,E,G, the total deformation trend of the bionic landing mechanism models is like that under uniform load, with deformation increasing gradually from the bottom of the suction cup to the frame linkage. The maximum deformation position is located on the frame linkage of all models, with maximum deformations of 0.12 mm, 0.54 mm, 0.16 mm, and 0.76 mm, respectively. It demonstrated that the bionic landing mechanism has good structural rigidity. From Figure 8B,D,F,H, the maximum stress of Model 1 is located at the connection between the tibial motor and the femoral linkage, with the maximum stress of 89.47 MPa; the maximum stress of Model 2 is located on the frame linkage, with a maximum stress of 31.68 MPa; the maximum stresses of Model 3 and Model 4 are located at the connection between the main arm and the femoral linkage, with maximum stresses of 15.79 MPa and 11.29 MPa, respectively. Under the same bending moment, the total deformation of Model 1 is smallest, while Model 4 has the largest total deformation. The total deformation of Model 1 is 78.13% smaller than Model 2, 27.45% smaller than Model 3, and 84.62% smaller than Model 4. About equivalent stress, Model 4 is the smallest, while Model 1 has the largest equivalent stress. The maximum stress of Model 4 is 87.38% smaller than Model 1, 78.13% smaller than Model 2, and 27.45% smaller than Model 3.

It can be concluded that the total deformation values of the four models are not much different. However, the equivalent stress of Model 4 is the smallest. It shows that the hollow PLA group can provide the best adaptability to bending resistance performance.

#### 4.4.3. Twist Moment

A twist moment equal to the bending moment is applied to the top of the bionic landing mechanism model. The twist moment *M_t_* exerted on the model is calculated as:

(7) $$M_{t}=M_{b}=0.177\;N\times 0.05\;m=0.00885\;N\cdot m$$

Displacement constraints are applied to the body of the bionic landing mechanism finite element model, restricting all degrees of freedom. The results are shown in Figure 9. From Figure 9A,C,E,G), the total deformation of the bionic landing mechanism models increases gradually from the bottom of the suction cup to the frame linkage of all models. The maximum deformation of Model 1 is located on the right edge of the frame linkage, with a value of 0.18 mm. For Model 2, Model 3, and Model 4, the maximum deformation is located on the left edge of the frame linkage, with values of 0.72 mm, 0.20 mm, and 2.20 mm, respectively. From Figure 9B,D,F,H), the maximum stress of Model 1 is located at the connection between the second arm and the actuating rod, with a maximum stress of 59.53 MPa. The maximum stress of Model 2 is located on the connection between the frame linkage and the femoral linkage, with a maximum stress of 133.83 MPa. The maximum stresses of Model 3 and Model 4 are located on the connection between the tibial linkage and the femoral linkage, with maximum stresses of 37.23 MPa and 35.28 MPa, respectively. Under the same twist moment, the total deformation of Model 1 is smallest, while Model 4 has the largest total deformation. The total deformation of Model 1 is 74.89% smaller than Model 2, 7.83% smaller than Model 3, and 91.77% smaller than Model 4. About equivalent stress, Model 4 exhibits the smallest maximum stress, while Model 1 exhibits the largest maximum stress. The maximum stress of Model 4 is 40.73% smaller than Model 1, 73.64% smaller than Model 2, and 5.24% smaller than Model 3. It can be found that the total deformation values of the four models are all within the controllable range. However, Model 4 has the smallest equivalent stress. It shows that the hollow PLA group can provide the best adaptability to torsional resistance performance.

From the above, it can be concluded in the structure group that hollow structures have better mechanical properties. Specifically, the equivalent stress value of hollow carbon fiber (Model 3) is 61.65% lower than solid carbon fiber (Model 1), and the equivalent stress value of hollow PLA (Model 4) is 71.33% lower than solid PLA (Model 2). It can also be concluded in the material group that PLA materials have better mechanical properties. Specifically, the equivalent stress value of hollow PLA (Model 4) is 20.73% lower than that of hollow carbon fiber (Model 3), while the equivalent stress value of solid PLA (Model 2) is 6.07% higher than that of solid carbon fiber (Model 1). Therefore, the final design choice of the bionic landing mechanism is the hollow PLA group (Model 4).

#### 4.4.4. Mechanical Characteristics of the Attachment Mechanism

**Figure 10:**
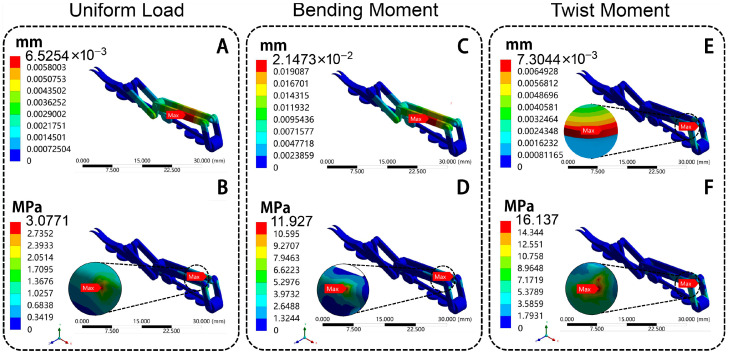
The structure statics analysis of attachment mechanism of bionic deployable landing mechanism. (**A**,**B**) Uniform load; (**C**,**D**) bending moment; (**E**,**F**) twist moment; (**A**,**C**,**E**) total deformation diagram; (**B**,**D**,**F**) equivalent stress diagram.

The attachment mechanism (hook-pad actuator of Figure 6B) of the bionic landing mechanism was separated, and its mechanical characteristics were analyzed, which can better explain the bionic advantages of this mechanism. The best attachment mechanism of the hollow PLA group was selected to test. The total deformation and equivalent stress of the attachment mechanism are shown in Figure 10.

Under uniform load, the maximum total deformation is located at the main arm, with a value of 0.0065 mm (Figure 10A). The maximum stress is located at the connection pins between the main arm and linkage 1, with a value of 3.08 MPa (Figure 10B). Under the bending moment, it can be observed that the position of the maximum total deformation is the same as those under uniform load. The maximum deformation is 0.021 mm (Figure 10C), and the maximum stress is 11.93 MPa (Figure 10D). Under twist moment, it can be observed that the position of the maximum total deformation is located at the connection pins between the main arm and linkage 1, with values of 0.0073 mm (Figure 10E) and 16.14 MPa (Figure 10F), respectively. Overall, the mechanical characteristics of the connection position between the attachment mechanism and the main deployable leg can be optimized in the future research. The stronger materials can be considered for replacement, and the dimensions of the position can be optimized to enhance structural mechanical performance.

### 4.5. Vibration Characteristics Analysis of Bionic Landing Mechanism

After structural optimization testing, a vibration characteristics analysis is conducted to verify the natural frequencies and mode shapes of the designed bionic landing mechanism, which are key parameters for addressing dynamic loads in the design.

**Figure 11:**
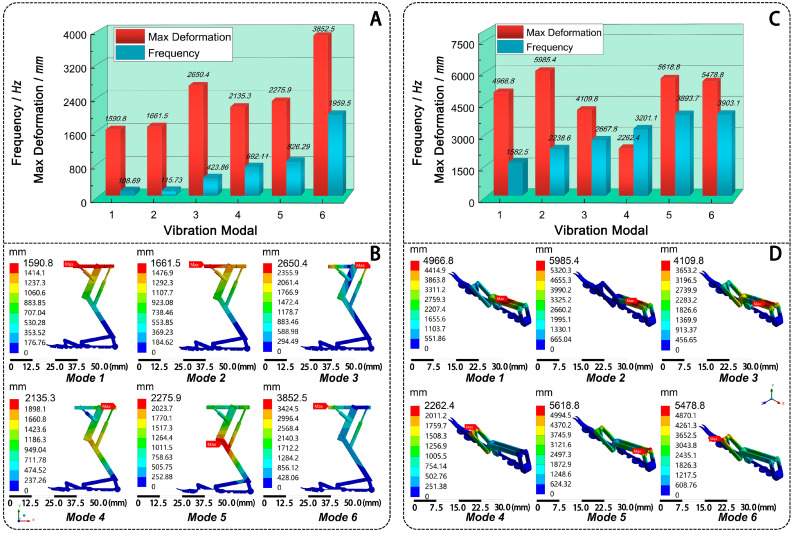
The vibration characteristics analysis diagram of bionic landing mechanism. The first six natural frequencies and max deformations of the landing mechanism (**A**) and the attachment mechanism (**C**); the total deformation mode shapes of the first six natural frequencies of the landing mechanism (**B**) and the attachment mechanism (**D**).

For the vibration characteristics analysis of the landing mechanism, the best hollow PLA group (Model 4) was selected to test. The constrained vibration performance analysis is performed using the finite element software Ansys 2022. The environmental temperature is 25°, and humidity is 40%. In the analysis, a fixed constraint is applied to the hook-pad actuator at the lower end of the mechanism, and the effect of prestress is not considered. Through finite element calculations, the first six natural frequencies of the structure are extracted, with the results shown in Figure 11A. The total deformation mode shape is illustrated in Figure 11B. All natural frequencies of the landing mechanism are above 108.66 Hz.

As shown in Figure 11B, the first mode primarily exhibits total bending deformation, with the landing mechanism swinging left and right around the hook-pad actuator and the maximum deformation occurring at the frame linkage. The second mode is characterized by overall bending deformation, with the landing mechanism swinging forward and backward around the hook-pad actuator, with the maximum deformation located at the left edge of the frame linkage. The third mode shows local bending deformation at the hinge between the femoral and tibial linkages, with the maximum deformation at the right edge of the frame linkage. The fourth mode is dominated by torsional deformation of the frame linkage around the hinge between the femoral and tibial linkages, with the maximum deformation still located at the right edge of the frame linkage. The fifth mode represents a higher-order torsional deformation, with the maximum deformation also occurring at the hinge. The sixth mode exhibits a global high-frequency bending-torsion coupled pattern, with the maximum deformation at the left edge of the frame linkage.

After analyzing the vibration characteristics of the whole landing mechanism, the vibration characteristics analysis of the attachment mechanism was further completed. The results are shown in Figure 11C,D). From Figure 11C, it can be observed that all natural frequencies of the attachment mechanism are above 1582.5 Hz, which is far greater than the operating frequency of the whole landing mechanism. Considering the high dynamic load performance requirements of the attachment mechanism, the high natural frequency can enhance stability and response speed during landing. From Figure 11D, the deformation of the six modes of the attachment mechanism is larger than the whole landing mechanism. The maximum deformation positions of the 1st, 2nd, and 3rd modes of the attachment mechanism are located at the main arm. The maximum deformation positions of the 4th and 6th modes are located at the connection pins between the end effector and linkage 2, while the maximum deformation position of the 5th mode is at the connection pins between linkage 1 and the main arm. Compared to the whole landing mechanism (Figure 11B), the attachment mechanism exhibits larger vibration deformation.

In summary, the first six natural frequencies of the bionic landing mechanism and the attachment mechanism are 108.69 Hz to 1959.5 Hz and 1582.5 Hz to 3903.1 Hz, respectively. Since the attachment mechanism’s natural frequencies are concentrated in the high-frequency range and differ significantly from the whole landing mechanism, the resonance phenomena will not occur. The results show that the bionic landing mechanism has better vibration characteristics and the ability to resist dynamic loads.

## 5. Conclusions

Based on the thoracic legs structure of *S. femorata*, a hook-pad cooperation bionic deployable landing mechanism model was designed for bionic FWMAV. Firstly, the 3D reconstruction models of the beetle’s three thoracic legs were built by the micro-CT system. The results show that the whole leg structure of *S. femorata* is hollow, with the femur being large and strong, the tibia being smooth and slender, the tarsomere composed of suction pads and bristle pads, and the fore tarsus being a bifurcated claw. The beetle leg’s hook-pad actuator is composed of tarsomere and fore tarsus. During landing motion, the hook and pad work together to ensure a stable landing, which can enhance adaptability to complex surfaces. From the analysis of the leg joint, the average length ratio of the femur, tibia, tarsomere, and fore tarsus is 5.64:4.79:0.79:0.86:1.15:1.90, and the average diameter ratio is 1.94:0.58:0.35:0.36:0.36:0.33. The femur is the longest, and the length of each tarsomere segment gradually increases. A comparison of the foreleg, midleg, and hindleg shows that the hindleg is the largest. The length and average diameter of the hindleg’ femur are about 2 to 3 times that of the foreleg and midleg, respectively. The length and average diameter of the hindleg’ tibia are about 1.6 times that of the foreleg and midleg, respectively, and both the femur and tibia have similar dimensions. The length of each tarsomere varies little among the foreleg, midleg, and hindleg, while the average diameter increases progressively from foreleg to midleg to hindleg.

On this basis, three key bionic elements were extracted, including the multi-tarsomere structure, the hollow tube structure, and the hook-pad cooperation structure. A bionic deployable landing mechanism was designed by a spatial multi-link mechanism.

This bionic landing mechanism can land on various contact surfaces, such as horizontal surfaces, curved surfaces, vertical surfaces, inclined surfaces, and object grasping. Then, the motion simulation analysis shows that the landing mechanism operates smoothly, and it can land in some complex environments. Additionally, the bionic design enhances flexibility and environmental adaptability. After that, four comparative models were established to analyze the influence of material and structure changes on the mechanical properties of the bionic landing mechanism, including solid carbon fiber (Model 1), solid PLA (Model 2), hollow carbon fiber (Model 3), and hollow PLA (Model 4). The results showed that Model 4 has the best mechanical performance. It meets the requirements of stability and deformation resistance during landing. The vibration performance analysis shows that the first six natural frequencies of the whole mechanism range from 108.69 Hz–1959.5 Hz, the maximum deformation range is 1590.8 mm~3852.5 mm, and the first six natural frequencies of the attached mechanism range from 1582.5 Hz–3903.1 Hz. Strong overall vibration characteristics and dynamic load resistance. Moreover, the attachment mechanism’s natural frequencies are concentrated in the high-frequency range and differ significantly from the whole landing mechanism. The results confirm that the bionic landing mechanism has better vibration characteristics and the ability to resist dynamic loads. Compared with the existing FWMAVs landing technology, the hook-pad cooperation bionic landing mechanism can land on different contact surfaces by combining grab, adsorption, and hook and claw actions, greatly improving the environmental adaptive ability. This research is expected to provide valuable technical insights for the design of landing mechanisms for bionic FWMAVs in diverse environments. In the future, FWMAVs equipped with this landing mechanism may be able to replace bees for precision pollination in agriculture or replace rotor-wing drones for indoor secret reconnaissance in the military.

## Figures and Tables

**Table 1:** The joint length and average diameter of the thoracic legs of *S. femorata*.

Joint		Tarsus	Tibia	Tarsomere			
Thoracic Legs				First Tarsomere	Second Tarsomere	Third Tarsomere	Fore Tarsus
Foreleg	Length/mm	4.10	3.82	0.74	0.88	1.10	1.62
	Diameter/mm	1.08	0.47	0.24	0.24	0.27	0.26
Midleg	Length/mm	4.62	4.12	0.79	0.91	1.20	1.85
	Diameter/mm	1.39	0.51	0.30	0.31	0.29	0.25
Hindleg	Length/mm	8.21	6.45	0.62	0.79	1.15	2.25
	Diameter/mm	3.35	0.78	0.51	0.52	0.52	0.49

## Data Availability

The datasets generated during and/or analyzed during the current study are available from the corresponding author on reasonable request.
